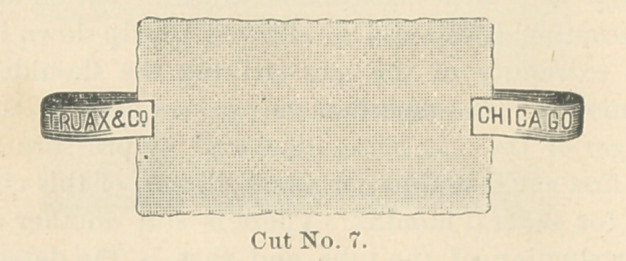# Chicago Medical Society; Stated Meeting of January 17th

**Published:** 1887-03

**Authors:** 


CHICAGO MEDICAL SOCIETY.

*Stated Meeting January 17, 1887.*—THE PRESIDENT, EDMUND J. DOERING, in the chair.

DR. LYMAN WARE read a paper entitled

A CLINICAL STUDY OF GLAUCOMA.

The author briefly referred to the history, pathology and etiology of glaucoma, and expressed his belief in the curative power of Von Graefe's operation of iridectomy. Unmistakable symptoms of glaucoma are supraorbital and ciliary neuralgia, increased ocular tension, periodoc diminution of vision, the appearance of a halo around artificial lights, a sluggish and widely dilated pupil and a shallow anterior chamber. Although increased tension may be associated with other diseases of the eye, its presence should always lead to a critical examination. Several cases were detailed: Mrs. M., aged 50, while riding in an open street-car, contracted a severe cold, which was followed by neuralgia over both eyes. The pain was so intense that she was at times delirious. Her weight was reduced from 110 to 80 pounds. She became entirely blind. On account of the eye being small and deeply set and the anterior chamber very shallow, sclerotomy was advised and performed. This gave immediate relief, but the pain returned again in a few days. When Doctor Ware saw the case the anterior chamber was almost obliterated, the glaucomatous lens pressed the iris forward until it came in contact with the cornea. With a Von Graefe cataract knife a free sclero-corneal incision was made and a portion of the iris excised, and the lens removed from both eyes. The pain greatly subsided, but the sight was irrecoverably lost. Another case was a man æt. 52 years, who complained of having had a pain in and over the left eye for five or six months, and had seen the halo about street lights. On examination tension was found increased, vision diminished one-half. Some months later iridectomy was performed, and a solution of eserine (4 grs. to the oz.)

instilled every four hours into the other eye. Pain was at once relieved and all symptoms of glaucoma rapidly disappeared. Mrs. M., aged 45, had frequent attacks of neuralgia, and noticed defective vision in the left eye six months before coming under observation. Tension was found much increased; she had seen halo about artificial lights for eight or ten months. Distant objects could be clearly perceived by right eye. Iridectomy was performed on the left eye with a view of abating the pain and preserving the vision of the right eye. The relief from pain was great, vision of the right eye fully restored and left eye much improved.

PROFESSOR E. L. HOLMES reported

A CASE OF FOREIGN BODY IN THE ANTERIOR CHAMBER.

The case was of special interest to him on account of the difficult diagnosis and the difficulty attending the removal of the foreign body. The doubt regarding the diagnosis arose from the fact that there was a small central perforation of the cornea. Below this, and not connected with it, was a fine greyish line extending downward and inward (R. eye), apparently in the substance of the cornea, fairly into the angle of the iris and cornea. This resembled the channels left after the removal of fine slivers of grain stalk which are sometimes thrown into the cornea obliquely from threshing-machines. The patient explained the accident as follows: He was setting a machine punch so that the punch would accurately fit the die. The power was applied, when the punch did not accurately correspond to the die. At the same instant something entered the patient's eye. Patient was first seen twenty-four hours after the accident with the eye slightly red, but not painful. Atropine was prescribed and he was sent to the hospital. The next morning the pupil was round and fully dilated. The media were all clear. The doctor felt great anxiety in regard to the case, but inasmuch as during five days there were no symptoms of inflammation along the supposed track in the cornea, he finally became convinced that there was a fine splinter in the interior chamber, in close contact with the cornea.

He made quite a long incision through the lower border of the cornea, the knife touching and moving the piece of steel, and endeavored by means of a fine forceps to seize the lower part of the steel and disengage the point from the tissues at the angle of the iris and cornea by carrying the steel farther towards the pupil. This was found to be impracticable, since the lower end of the piece was firmly held by the tissues. He used all the violence considered warrantable. As the anterior chamber was without aqueous humor, there was some difficulty in further procedure without violence to the lens or iris. He consequently extended the wound in the cornea upward so the upper end would correspond with the upper end of the steel. This end was easily seized and with considerable force withdrawn. The splinter was three-sixteenths of an inch in length. Eserine at first, then atropine with antiseptic dressings were applied with great care. There was no reaction, the patient recovering perfect sight, except as far as there was dimness from the central cicatrix in the cornea. Vision was good five weeks after the patient had returned home.

PROFESSOR HOLMES also reported

A CASE OF INTRA-OCULAR TUMOR.

This tumor, filling the sclerotic, is a sarcoma of the choroid. The patient, a man 62 years of age, had been under the observation of several specialists during the past year, but could not give me a definite expression of their opinion. When first seen, a few days ago, the cornea was perforated and presented a staphylomatous projection of the growth. For a year there has been pain and for the last six months very great pain. The tissues of the orbit around the globe were greatly swollen, but not indurated. In enucleating the eye he expected to find the sclerotic destroyed posteriorly and the orbital tissues invaded. The enucleation, however was performed as easily as in ordinary cases. The optic nerve is seen to be enlarged several millimetres behind the sclerotic. The swelling in the orbit was caused by nodules of fat filled with numerous blood-vessels. Dr. Ochsner pronounces the tumor to be a small round-celled sarcoma with very little pigment. The nodules of fat are free from sarcoma cells. This class of tumors, if removed early, are not very liable to return in the orbit. They may, however, reappear, especially in the liver or other internal organs. They must, consequently, be regarded as quite malignant.

DR. BOERNE BETTMAN read a paper on the

CONNECTION BETWEEN OCULAR AND NASAL DISEASES.

The author thought that numerous pathological conditions of the eyes and lids are attributable to abnormal changes in the nose, and that in these cases treatment of the ocular organs alone will fail to alleviate the trouble. After referring to Hack's monograph on the subject, Dr. Bettman detailed several cases in substantiation of his theory: A boy of 10 applied for treatment of epiphora of both eyes. The eyes were constantly weeping. An examination of the nose revealed an extensive swelling of the anterior portion of both turbinated bones. When these parts were touched with the probe profuse lachrymation was induced, and a light thrown into the eye by means of the ophthalmoscope produced violent sneezing. A deep incision was made in the swelling with a knife electrode, and a flat burner was also employed. The slough was completely thrown off in fourteen days and the boy cured in one month, the eyes receiving no treatment. Polypi of the nose have been found to produce secondary affections of the eye. Hermann S. was prevented from following his trade of a cabinetmaker on account of the excessive flow of tears; he also complained of pain in the eyes. Polypi were removed from the middle turbinated bone with the Jarvis snare and a cure effected. E. B., aged 16, was extremely sensitive to light, and the eyes were both bathed in tears. Each time the eyes were exposed to a glare of light she sneezed violently. There was Hack's swelling in both nostrils. Two pledgets of cotton were soaked in a 5 per cent. solution of cocaine and allowed to remain five minutes at a time. There was an immediate effect, and in three-quarters of an hour she was able to bear the light. The patient refused cauterization and employs cocaine to avert photophobia. The majority of cases coming under Dr. Bettman's observation have been treated by applications of the galvano-cautery to the nasal mucous membrane. The applications restricted to the anterior end of the turbinated bone frequently fail to give relief. It has been found that a sensitive area exists at the posterior end of the inferior turbinated bone and also at the anterior part of the nasal cavity, in the angle forming the boundary of the vestibule. In conclusion, the author thought oculists should always subject the nose to a thorough examination when seeking the source of ocular complaints.

DR. H. M. STARKY read a paper entitled

SOME MODIFICATIONS IN THE TREATMENT OF STRICTURE OF THE NASAL DUCT.

The author said that about 1883 the Western Suppository Co. made a lachrymal bougie of medicated gelatine of such elasticity that it could easily be passed into the nasal duct. It was less painful than a metal probe, and its slow solubility kept the mucous membrane at the point of stricture distended so that it could be acted upon by the medicine from thirty to sixty minutes. He thought results showed the use of electrolysis in these cases to be often unsatisfactory. The object to be attained is to restore the diseased parts to as nearly a normal condition as possible, and the most satisfactory treatment is by using injections more and probing less frequently. The author determined to try the effect of probing the punctum without slitting the canaliculus, followed by astringent injections over the inflamed surface. This treatment proved entirely satisfactory, and in about five weeks a patient went to his home in another state with apparently perfect recovery, and no destruction of tissue.

The following case was given as illustrating the author's method of treatment: Mrs. L. suffered from lachrymation of each eye for two years. There was severe lachrymal conjunctivitis of the right eye, the punctum being contracted one-half. On dilating the right punctum a No. 2 probe could be passed without difficulty, but the whole interior of the nasal duct had the peculiar velvety feeling that is caused by thick villous mucous membrane. The same condition, in less degree, was found on the left side. Treatment was commenced by applying a weak astringent and washing out the lachrymal canals thoroughly each day with boric acid lotion, followed by a weak astringent. Once a week a probe was passed through the dilated punctum down to the naris, using a larger probe each time until No. 7 was reached. The result was satisfactory, and in six weeks the patient returned home apparently well.

PROFESSOR W. FRANKLIN COLEMAN read a paper on

SYMPATHETIC OPHTHALMIA.

Disease in the sympathetic eye generally occurs when there has been a wound or operation in the dangerous zone of the diseased eye. Becker, in 1875, collected twenty-two cases of sympathetic ophthalmia from cataract operations, foreign bodies lodging in the eye, and degeneration of a lost eye, or other causes. Doctor Coleman read in detail the clinical history of the disease, and enumerated the causes, histories and results of the treatment of a large number of cases. In regard to treatment he advised as follows:

| CONDITION DISEASED EYE. | CONDITION SYMPATHETIC EYE. | TREATMENT. |
|---|---|---|
| Blindness. | Normal. | Enucleation in unintelligent and children. |
| Blindness. | Sympathetic irritation. | Enucleate. |
| Blindness. | Sympathetic inflammation. | Enucleation not often advisable. |
| More or less vision. | Normal. | Do not enucleate generally. |
| More or less vision. | Sympathetic irritation. | Better enucleate. |
| More or less vision. | Sympathetic inflammation. | Do not enucleate. |
| Acute ophthalmitis. | Normal. | Never enucleate. |
| Acute ophthalmitis. | Sympathetic irritation. | Puncture and foment diseased eye, then enucleate. |
| Acute ophthalmitis. | Sympathetic ophthalmitis. | Treat ophthalmitis, and then enucleate. |

PROFESSOR F. C. HOTZ said: He thinks the theory of the author in regard to the closing of Schlemm's canal and the approximation of the iris to the cornea interfering with filtration cannot account for glaucoma. Pathological anatomy has so far failed to find the cause, and we have to rely on clinical studies to build up a theory which will account not for the late stage, the fully developed glaucoma, where the sight of the eye has been permanently destroyed by the disease, and which the pathologist gets from the oculist after enucleation, but for the first stage, the premonitory symptoms before it becomes an acute attack; a stage which the pathologist has not yet investigated with his microscope. At that stage who can say certainly what glaucoma is? It is probable that various causes lead to the same result. He believes that the agglutination of the iris to the cornea, the compression of Schlemm's canal or any other part of the eye, are consequences, and are not primary causes of glaucoma. He was somewhat surprised that, in a paper addressed to general practitioners, the author attached so little importance to the clinical symptoms in glaucoma, of a general character, such as gastric and febrile disturbances in connection with hemicranic headache. These symptoms often cause the practitioner to fail to discover glaucoma. He recalled a number of such instances. Last October a lady came under his care who had been under the treatment of a physician for four or five weeks for malarial fever and dyspepsia, which was the beginning of an undoubtedly characteristic and typical attack of glaucoma. But the attending physician's attention was attracted by the coated tongue, the nausea, vomiting, severe headache and excited pulse, and he treated the patient for these daily attacks of headache, while he diagnosticated malaria, and used antiperiodic remedies, utterly disregarding the condition of the eye, although the sight was at first nearly extinguished, and only returned to a certain extent after the attack lost somewhat its severity. Another case: A poor woman lost one eye from glaucoma ten years before; the eye was blind and hard, showing the characteristic state of an eye in which glaucoma had run its course. She was attacked by a severe pain in the head extending over the left side, could not sleep for several weeks, was nauseated, vomited, and showed symptoms of some general disturbance. The physician treated her for the stomach trouble and headache, and although she told him time and again that her sight was getting poor, and suggested that an oculist had better examine her eye, he paid no attention to this, and the result was that two months after this attack the sight was entirely gone and could not be restored. In still another case both eyes were neglected until the patient could perceive only a little flicker of light, before it was considered necessary by the attending physicians to pay any attention to the eyes. Dyspepsia, gastric fever, malaria, and sick headache were the diagnoses, and the treatment was in accordance. He thought it well to bring out these points, and to call the attention of every physician to the fact that such attacks sometimes mean something more serious than a disturbance of the stomach, and that when the patient, during such attacks, speaks of the eyes as being troublesome, or the sight as becoming dim, it is worth while to pay attention to it, and to remember that acute glaucoma is often ushered in with these general constitutional symptoms.

Dr. Lyman Ware said, he had only a word to say about the disturbance of the equilibrium of secretion and excretion. It has been fully demonstrated that it is only by restoring the equilibrium that sight is saved. He quite agrees with Doctor Hotz regarding febrile symptoms, but it is his experience that they are *secondary* rather than *primary*.

Dr. Henry Gradle said: The cases which Dr. Bettman presented are of great interest from the fact that they have only lately been recognized. Dr. Gruening, of New York, was the first to point out that there existed affections apparently of the eye, but which in reality originated from the nose. He has watched for these cases ever since Gruening's paper first appeared, and would say that the cases in which the nasal trouble is *entirely* the cause of eye disease are not very frequent. But he had seen instances where affections of the eye were certainly complicated by nasal trouble, and the nasal trouble prolonged the eye disease. He recollected a number of cases of eye disease either kept up or originated by nasal trouble. The first of these is a pseudo-erysipelas of the lids, which is not an infectious disease, but merely a secondary affection of the blood-vessels, only resembling erysipelas clinically. It is entirely due to irritation and engorgement of the blood-vessels in the front part of the inferior turbinated bone. A second type of nasal affection giving rise to eye trouble is true periodical hay fever, and a non-periodical irritability of the nose, resembling hay fever. He has published four cases, and has since seen another, of periodic conjunctivitis characterized by the formation of granules and follicles, which trouble always receded in winter, to reapper again in the spring and summer. In two of these cases a diagnosis of hay fever has since been made. He has seen a case which had been treated for trachoma by a number of specialists, where the history of the nose showed that the affection was of nasal origin. The same trouble may exist in a non-periodic form, and present all the symptoms of hay fever, the trouble not being limited to any season, but occurring in any part of the year, lasting a few days or weeks. But these cases are not common. In one of these cases he was able to effect a complete cure by cauterization of the nose. A third type of nasal affection giving rise to ocular symptoms is true catarrh of the upper and front part of the mucous membrane of the nose, where the membrane is distinctly reddened and where there are generally slight and by no means prominent symptoms of catarrh. In these cases he has very frequently found troublesome epiphora without any stricture of the duct; in some cases the test was made by using delicate probes. Such cases are entirely curable by simple treatment of the nose. He has found a not sharply defined case of asthenopia, due not entirely to the nose, but complicated with refractive trouble where nasal treatment was necessary to complete a cure. Once or twice he has seen polypi play the same role, and a number of times found the starting-point of the irritation not in the front of the nose, but in the posterior part, in the form of the common adenoid vegetations.

This is a subject which has not been fully dealt with in literature, but he has several cases where the extirpation of the large post nasal tonsil has given decided relief to the eye. Then he has found that in a few cases ulcers or chronic inflammation of the cornea were kept up by nasal trouble which was probably started in the first place by a copious flow of tears from the eye. He has observed that local treatment by means of calomel, atropia, and the customary applications to the eye, proved inefficient, while the addition of nasal treatment hastened the cure of some of these tedious cases. The nose was probably normal to start with, but the continued flow of tears produced either small erosions or some little catarrhal troubles of the mucous membrane at the front of the nose, subsequently increased to chronic catarrh, leading to congestive obstruction of the tear passages, or exerting an unfavorable nervous influence upon the eye trouble. Finally, as a rare instance, he mentioned one case which is now cured. The patient was sent to him for polypi, which, however, proved to be the minor trouble in the nose, the real trouble being an immense vascular tumor occupying the entire floor of the right side of the nose, covering the inferior turbinated bone and reaching about to the middle turbinated bone. The patient had been reduced in strength, and the slightest exertion on his part produced hæmorrhage, therefore the most careful operative procedure was necessary. He finally succeeded in removing the entire tumor by the galvano-cautery in twenty sittings. As the tumor began to shrink the hæmorrhage was less, but he lost thirty or forty ounces of blood in six weeks. During the latter part of the treatment his right eye began to bulge, and he complained of double sight. It has remained healthy, but there was an unmistakable development of vascular tissue in the orbit and behind the eye, which receded by the time the tumor had been extirpated from the nose.

DR. BOERNE BETTMAN said that he was very glad to hear Dr. Gradle corroborate his statements. He is well aware that these cases are comparatively rare; although he has recorded in his case book about twenty, seen during the last two years. He is acquainted with the article published by Gruening. His attention was first called to the subject by the work of Hack, and since reading that he has made it a point never to allow an eye patient to leave his office until his nose has received a very thorough examination. He has seen a number of cases such as mentioned by Dr. Gradle, but thought it better to describe to-night only the typical ones. The connection between ocular and nasal troubles is a point all oculists should bear in mind, and when they find no local cause for epiphora they should examine the nose.

DR. A. P. GILMORE said he would like to add one word in regard to glaucoma, and that is, that the importance of tension did not seem to him to have been sufficiently dwelt upon. Any careful general practitioner can ascertain whether the tension is increased or not, simply by comparison with the tension of his own eye. All pain referred to the eyeball, with or without the accompanying neurotic symptoms mentioned in the paper, does not mean glaucoma. Unless there is increased tension you cannot diagnosticate glaucoma. The author does not mention Badal's operation in the treatment of glaucoma. It is certainly entitled to a place among the operative measures. He would only speak of one point in Doctor Starkey's paper, viz., epiphora. He does not believe, with many, that epiphora is due primarily to a stricture which prevents the escape through the nose of the natural amount of fluid secreted, but is due rather to reflex irritation causing an hypersecretion of tears. In health the eye is moistened with a moderate secretion. When the lachrymal gland is removed the eye continues to be moist and the cornea retains its lustre. Tears are not essential to the lubrication of the eyeball; their function is to protect the eye against foreign bodies. A bit of dust under the lids will cause profuse lachrymation and the tears will flow over the face, not because of an obstruction to the natural amount of fluid secreted through the natural passage, but because of a hypersecretion due to reflex irritation. For treatment he never uses a probe larger than Bowman's No. 6, usually No. 4. He seldom finds it necessary to make Bowman's operation in epiphora. He thinks its use is unnecessarily frequent. He uses astringent and antiseptic solutions with a syringe small enough to be easily introduced into the puncture when slightly dilated. He is very careful to treat any nasal complications; it is impossible to treat diseases of the eye successfully without recognizing and treating reflex irritations of the nose.

DOCTOR STARKEY said that his paper was necessarily cut down very much. As first written he had given some space and attention to cases similar to those mentioned by Dr. Bettman. He had also spoken of the probability that in many cases of epiphora, where there had been inflammation of the tissues lining the lachrymal canals with partial closure, a continual irritation of the canal in some way, perhaps reflexly, so stimulates the lachrymal gland that the tears are poured forth more abundantly. There are well-known cases where the lachrymal canals have been completely closed by injury or operation, and yet lachrymation is not annoying, although the gland has not been extirpated; tending to show, as mentioned by Doctor Gilmore, that the normal secretion of tears is ordinarily very limited. It seems to him that in many instances lachrymation is due to irritation propagated reflexly, and therefore in treating such cases he thought of trying to restore the mucous membrane of the lachrymal canals to the normal condition, as well as to look for and treat points of irritation elsewhere.

PROF. J. E. COLBURN said: In case of injury where there is danger of sympathetic irritation, a foreign body being lodged in the anterior chamber, iris, ciliary body, or the choroid, where the chances are that in order to give all the advantages of treatment the patient must necessarily be idle for a considerable length of time, and where the sight in the injured eye has been irretrievably lost, he thinks it advisable to make the operation of evisceration or abscission as early as possible. The patient, if a laboring man, is then relieved from a long enforced idleness and anxiety, and the danger that lack of care frequently causes in this class of cases. Where the appearance is first to be considered, and the patient can be constantly under observation, the operation can be postponed, but with the strict injunction the patient is to be under constant surveilance. In a large majority of cases where there is great damage done and the foreign body is out of sight, it is safe and advisable to make the operation, trusting to that to save the other eye. In a case that came under his observation recently a piece of steel entered the anterior chamber near the centre of the cornea, passed through the iris and lodged in the sclera. No operation was performed, and the fellow eye became sympathetically affected, and on account of its sympathetic disturbance had to be removed. The steel produced some local irritation, and the eye was caught and rolled strongly toward the nasal canthus, and the piece of steel was found projecting into the orbit from the sclera and was removed. The track of the steel through the sclera was surrounded by a large mass of fatty degeneration, which was also removed. Vision remained about one half.

PROF. W. FRANKLIN COLEMAN said he agreed with Doctor Colburn as to the desirability of timely enucleation in the case of a laboring man to save his time, but should ophthalmitis set in he should not, under any circumstances, enucleate the eye. He believed it is rare for German operators to risk removing an eye in a case of ophthalmitis, but in England they scarcely hesitate to remove an eye under any circumstances. He had never regretted recommending a patient to have an eye enucleated, but he sometimes regretted that he did not urge the patient to have the eye out in order to avoid the fearful risk of sympathetic inflammation. He is astounded at the position of so eminent an authority as Noyes who says, "I hesitate to enucleate the eye on account of appearance, and do not do so unless symptoms of irritation or inflammation appear which I cannot relieve with medical treatment." In nineteen out of twenty cases the lost eye is not worth saving, but is a blemish, and an artificial eye would be more ornamental. And if a man wishes to get work he will deceive the very elect as to which is the real and which the artificial eye. He cannot see any advantage in not advising enucleation where the eye has been injured to such an extent as to menace the fellow eye.

PROF. GILMORE asked Prof. Holmes why he did not try a magnet.

PROF. HOLMES replied that he had been in so much doubt what to do that he thought best to first try incision and forceps. He did not believe the best magnet could have liberated the end of the steel, buried in the tissues of Fontana's space. It is remarkable that so long a piece of metal could have been thrown through the cornea, making so minute an opening, and lodged in the anterior chamber, as described, without injury to the iris or lens.

PROF. COLEMAN said to his mind the magnet in the eye is a delusion and a snare. For instance, if you introduce a magnet within the eye not knowing where the foreign body is before placing the point of your magnet, you have to search the whole cavity of the eyeball and reduce it to a jelly before you can extract the body. Granted no great harm is done if you do not extract it with the magnet, for you can afterwards enucleate the eye. But so far as he has tried it, and has seen others experiment with the magnet, it does not give satisfaction.

PROF. HOLMES replied: That is very true in many cases where the steel cannot be seen with the ophthalmoscope, but he thinks where a view of the foreign body can be obtained early, the magnet may be employed with brilliant results. There are now so many cases reported with excellent results after extraction with the magnet, that he cannot think it a delusion and a snare by any means.

PROF. COLBURN said he recently saw an interesting case in which the foreign body was lodged about half way between the ciliary body and the entrance to the optic nerve. The operator cut through the sclera about where he thought it was lodged, passed the magnet in and brought out the foreign body apparently without wounding the retina at the point of attachment. The patient made a good recovery.

### CHICAGO MEDICAL SOCIETY.

*Stated Meeting, February 7, 1887.*—THE PRESIDENT, EDMUND J. DOERING, M.D., in the chair.

PROFESSOR W. E. QUINE, Chairman of the Committee appointed to convey to N. S. Davis, M.D., L.L.D., a formal expression of the Society's estimate of his labors and character, prefaced the presentation of the report by a few well chosen words of congratulation to Prof. Davis on his arrival at the fiftieth anniversary of the date on which he received his diploma to practice medicine. The report of the committee, which had been engrossed on parchment, was then read and presented to Doctor Davis, who responded in a feeling manner. The resolutions were as follows:

"*Resolved*—1. That N. S. Davis, M.D., LL.D., ex-President and only surviving charter member of this organization; ex-President of the Illinois State Medical Society; twice President of the American Medical Association; President of the Ninth International Medical Congress; Dean of the Faculty of the Chicago Medical College, etc., etc., is the acknowledged founder of the American Medical Association, and practically the founder of all medical organizations on this continent. His labors in this direction have been of incalculable benefit to his profession and the community at large.

"2. That he has been one of the most earnest and influential workers of his time in the direction of improving the methods, increasing the thoroughness, and enlarging the scope of instruction in the medical colleges of this country, and has thus contributed vastly to the elevation of the average scholarship of his profession.

"3. That he has been one of the most devoted and eminent of the medical teachers this country has produced; one of the most industrious and powerful of the medical writers and orators; one of the most active and conscientious of the original investigators; and one of the most acute and philanthropic of the medical practioners. He has received and merited the highest honors which the medical profession had the power to confer, and he has been outranked by no man in point of popular confidence and esteem.

"4. That as a man he has made an inspiring record—a record of purity and dignity of character; of unyielding devotion to principle; of tireless zeal for the suppression of intemperance, and of such charity as made him pre-eminently the physician and benefactor of the poor, and led him habitually to subordinate opportunities for personal gain to impulses in the direction of making his fellow creatures happier and more useful. A born teacher of teachers! leader of leaders! exemplar of christian philanthropists! the Chicago Medical Society hails thee as the most illustrious of its members, and salutes thee as an honor to the profession and a blessing to thy race."

Signed by President E. J. Doering; Liston H. Montgomery, Secretary, and Dr. J. J. Angear, William E. Quine and James H. Ethridge, Committee.

PROFESSOR J. A. ROBISON read a paper on

THE CLIMATIC TREATMENT OF DISEASE, which was discussed at the meeting of the society on February 21st.

He said there is probably no field in therapeutics in which the general practitioner becomes so quickly lost as that of the climatic treatment of disease. He seldom has the time or opportunities to investigate the subject personally, and what little knowledge of the subject he possesses has been gleaned from the voluminous literature written by various authors on this topic. Even this knowledge is ill-defined. There is a great difference of opinion among writers on climatology as to what should be the altitude, temperature, dryness or moisture, etc., for the treatment of various pulmonary diseases. The purpose of this and following papers will be to formulate the desiderata for climates in the treatment of various pulmonary diseases.

Inasmuch as phthisis pulmonalis constitutes the largest class of these diseases, he noted what some eminent authorities say are the requisites in the climatic treatment of this disease.

From the opinions of these authorities we can tabulate certain facts :

1. The climate must be such as to insure *pure air free from dust, or poisonous germs.*

2. Such air is more apt to be found at an elevation of 1,000 feet, or more, above the sea level.

3. There should be an equable temperature, neither too warm nor too cold; the air should be in continuous motion and yet there should be no wind storms.

4. There should be plenty of sunshine.

5. The landscape should be pleasing.

6. The health resort should be easily accessible and home comforts with congenial society easily obtained.

7. The patient should be able to take almost daily outdoor exercise without fatigue.

When patients are able to find homes in climates which nearly fulfill all these conditions, clinical observations demonstrate that consumption may not only often be arrested but cured. It is a well-known fact in mycology that a modification of the environment often prevents bacteria from thriving and multiplying, and this may account for the improvement which often follows the residence of a consumptive in a pure climate where he can take exercise. Bodily nutrition is increased, the power of resistance to disease is augmented, and the germs of consumption die. The disappearance of the disease is heralded by the improvement of the appetite and the digestion, the increase in force of the circulation, the stimulation of the respiratory functions with increase of normal oxidation and bodily heat. Thus with the improvement of the general bodily nutrition is favored that condition of the lung where there is absorption of the inflammatory exudates present in incipient phthisis, or the formation of cicatrized tissue in the latter stages of the disease.

Having thus considered the climate conditions favorable for the treatment of consumption, he noted the claims which certain localities in the United States present as being suitable places to which to send consumptive patients, including Asheville, N. C., Marietta, Ga., and Lookout Mountain, Tennessee.

PROFESSOR F. E. WAXHAM exhibited

### MODIFIED INTUBATION INSTRUMENTS.

DR. WAXHAM said: About thirty years ago a new operation was proposed as a substitute for tracheotomy, by M. Bouchut, of France, and so great was the opposition to this new operation, which was styled tubage of the larynx, that a committee headed by Trousseau, appointed by the Academy of Medicine, reported adversely in regard to it, and the operation was so deeply buried in oblivion, that early operators in this country were not even aware of the attempts and failure of Bouchut.

The most earnest advocates of intubation do not consider that the instruments are perfect; indeed the operation is yet in its early infancy, and it may be years before the method is fully and perfectly developed. One of the chief objections to the operation, indeed the only valid objection, is the difficulty of swallowing, the danger caused by the falling of food and fluid into the bronchial tubes through the canula, and the too frequent occurrence of broncho-pneumonia. He would not exaggerate this danger, but certainly it is true that many patients die of broncho-pneumonia from this source. To overcome this difficulty he had had Messrs. Charles Truax & Co., of this city, modify the O'Dwyer tubes, by making them with smaller heads.

Cut No. 1—O'Dwyer Tube.

Cut No. 2—Waxham's Modified Tube.

The tube is prevented from slipping into the trachea, by a rubber collar. (Cut No. 3.) To this rubber collar is attached a flap, or artificial

Cut No. 3.

epiglottis. (Cut No. 4). During the act of deglutition the larynx rises

Cut No. 4.

and presses against the base of the tongue and the epiglottis, and the pressure of the epiglottis holds the rubber cap, or artificial epiglottis, over the aperture of the tube, thus preventing the dropping of solids into it, and as deglutition ceases, the larynx falls and the elasticity of the rubber throws it upwards. This rubber attachment does not entirely prevent the falling of liquids, of water particularly, into the tube, but it is of very great assistance in swallowing solids and semi-solids. He has used this modification in a number of cases with good results, and he had, at that time, a little patient convalescing from a desperate attack of diphtheritic croup, in which this modification was used.

He also presented a modification of the mouth-gag. (Cut No. 5.) In

Cut No. 5.

the old gag the extremities are liable to strike the shoulder, especially if the child is not held well and is allowed to slip down in the lap of the attendant, the extremity of the gag striking the shoulder and throwing it out of the mouth. The modified gag passes back of the head, and we avoid the danger of the gag being displaced by pressure of the shoulder. This gag was first suggested by Dr. McWilliams, of this city, and has been in the market for several months. There is still another danger that may follow the introduction of the tube, and that is the detachment of membrane below the tube, or the pushing of membrane down ahead of the tube when it is introduced. An accident of this nature had occurred to him recently: a tube was passed down into the larynx and the respiration at once ceased, the child turned blue and seemed upon the point of death. The tube was at once removed, but the respiration was only slightly improved and the tube was again introduced, with the same result. It was again removed and the trachea forceps (cut No. 6) that he had devised for this purpose, was introduced into the mouth, and a mass of membrane, a perfect cast of the trachea and the two larger bronchial tubes, removed. After the removal of this cast the tube was again introduced, and respiration was easy. Without these forceps an immediate tracheotomy would have been necessary.

Cut No. 6.

He then presented to the Society a membraneous cast from the trachea, larynx and bronchial tubes of the late Dr. Newton; the specimen being remarkable not only for its thickness and its extent, but for the rapidity of its growth; it was produced within three or four days after the invasion of the larynx. The fate of Dr. Newton, whose early death all regret most sincerely, teaches a sad lesson: it teaches the danger that besets the faithful physician, and the necessity of taking every possible precaution against the contraction of this hydra-headed monster, this justly dreaded disease diphtheria.

To those practicing intubation he advised that an ordinary rubber cot with the end cut off should be slipped over the forefinger, and then during the operation, if the gag is displaced, the finger is protected; as an additional protection, it will be well for the operator to use a respirator, (cut No. 7) an ordinary pad of antiseptic gauze with tape attached to

Cut No. 7.

secure it in place. This pad should be passed over the mouth and nostrils, and should be used by the physician when inspecting the throat or when operating upon a bad diphtheritic case. He regards it as a duty that every physician owes to himself, his family and friends, to take these precautions, especially in the treatment of bad diphtheritic cases.

PROFESSOR JOHN BARTLETT said: Doctor Waxham has given us the position of the O'Dwyer tube when *in situ*, indicating that the beveled facet on its upper extremity should present upwards and forwards. By several practitioners in this city the tube has been introduced contrariwise; that is with the bevel of the flange looking upwards and forwards. This position of the tube, it is maintained by those who prefer it, has an advantage, namely, that when it is so placed, the patient's ability to swallow is appreciably greater than when it is inserted as intended by Doctor O'Dwyer. The anatomist, Professor Hoadley, prefers this reversed position. He has now introduced the tube in this manner in eight cases, and in seven of these the patients, directly after the operation, could swallow fluids without the least difficulty. Doctor Hoadley maintains that the flange of the tube as placed by him deeply within the vestibule of the larynx, in no wise interferes with the functions of the epiglottis, or the aryepiglottic folds. Confusion in regard to the proper position of the tube, has arisen here from earlier cuts of the instruments accompanying some of the cases; these erroneously represented the tube placed with its longer margin forward. He desired to say a few words in regard to feeding these patients. There are reasons for believing, that, by the use of a French conde catheter of proper size and introduced some distance into the gullet through the nasal cavity, thorough alimentation could be secured without distressing the child, or, a suitable stomach tube might be introduced through a stomach-tube director without greatly distressing the patient. Stress might be properly laid upon the fact that pultaceous food, as bread soaked in milk, is swallowed more readily than water, or fluid nutriment.

PROFESSOR CHARLES WARRINGTON EARLE said, he thought Doctor Waxham, who is certainly the most successful in this operation, should warn the general practitioner who has had little experience against trying this operation without some one standing at his elbow to show him just how to perform it. There are few who do it well, and he knew of excellent surgeons in this city who may be trusted in everything else, but who have tried to do this operation and signally failed. And, although Doctor Waxham talks as if it is very easy, no person should try the operation for the first time without having some one by him who is particularly skilled. He remembered very well trying to perform the operation when Professor Waxham was present. He tried to introduce the tube but could not, and Doctor Waxham took the instrument and did it in two seconds. He thought one should not try to make it appear so easy.

PROFESSOR WAXHAM, in closing the discussion, said: He would say a few words in regard to feeding patients. One of the secrets of success is proper feeding, and the attending physician should superintend the feeding of the child. He may tell the people to feed it *bread* and milk, or semi-solids, and if he investigates the matter he will find they are giving it half a teaspoon of milk with a little bread, and the milk trickles into the trachea and the bread is rejected. If they are told to make a custard they make it so soft and fluid that it will *trickle* into the trachea. It is very important that the physician should superintend the feeding, personally. It is well not to give the child liquids; a small piece of ice placed in a piece of cloth held in the mouth will quench the thirst, and it should not be allowed to swallow for several hours, when it will swallow very much better than if the feeding is attempted at once. He had always made it a practice to introduce the tube with the beveled portion directed forwards and the projecting shoulder backwards, and regarded that as being one reason of his success. It seems a reasonable method of placing the tube because, if the higher portion is directed backwards the epigolottis can more perfectly close over the aperture of the tube than if the high portion is introduced forwards. Little or no difficulty is ordinarily experienced by the expert in removing the tube. It has been removed over and over again in two seconds; and yet, occasionally, if the tube becomes turned in the larynx, or if from the small size of the tube it sinks to the bottom of the larynx, it becomes difficult even for the expert to extract the tube, and in such a case after two or three careful attempts, we should give the child an anæsthetic so as to have it entirely quiet while the extractor is passed into the tube. In extracting the tube it is best to place the finger over the base of the tongue, when the child will gag and the tube will rise to the finger and it can be caught with the extractor.

## Figures and Tables

**Cut No. 1 f1:**
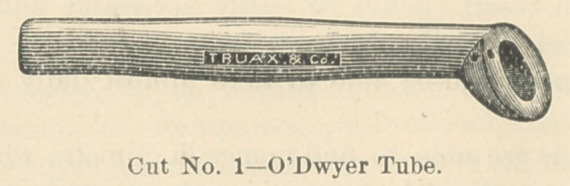


**Cut No. 2 f2:**
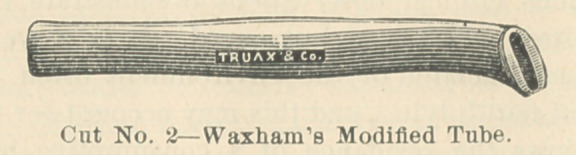


**Cut No. 3. f3:**
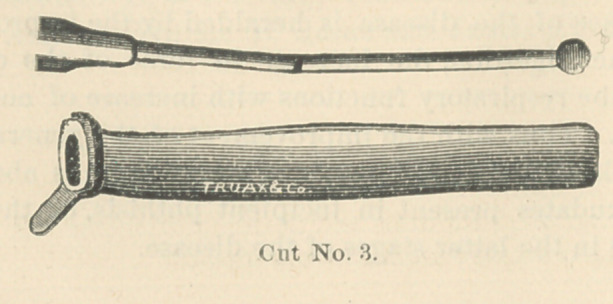


**Cut No. 4. f4:**
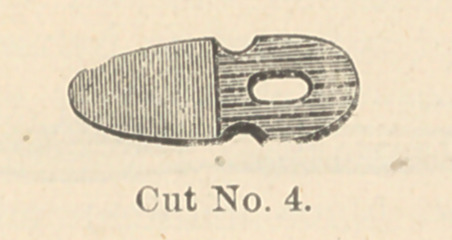


**Cut No. 5. f5:**
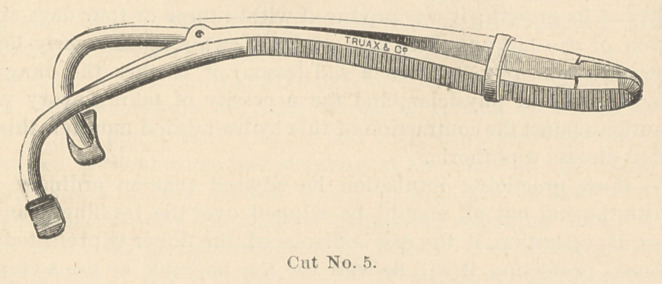


**Cut No. 6. f6:**
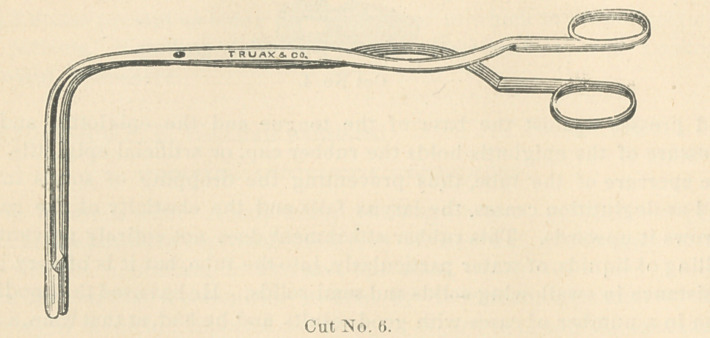


**Cut No. 7. f7:**